# Post-Traumatic Stress Disorder among the Flood Affected Population in Indian Subcontinent

**DOI:** 10.3126/nje.v9i1.24003

**Published:** 2019-03-31

**Authors:** Mohammad Asim, Ahammed Mekkodathil, Brijesh Sathian, Rajesh Elayedath, Rajeev Kumar N, Padam Simkhada, Edwin van Teijlingen

**Affiliations:** 1 Academic Research Associate, Clinical Research, Trauma and Vascular Surgery, Surgery Department, Hamad General Hospital, Doha, Qatar.; 2 Injury prevention coordinator, Clinical Research, Trauma and Vascular Surgery, Surgery Department, Hamad General Hospital, Doha, Qatar.; 3 Assistant Professor, Schoool of Behavioural Sciences, Mahatma Gandhi University, Kerala, India; 4 Director & Associate Professor, Schoool of Behavioural Sciences, Mahatma Gandhi University, Kerala, India; 5 Associate Dean for the Faculty of Education Health and Community, Professor of International Public Health, Liverpool John Moores University, UK; 6 Professor, Centre for Midwifery, Maternal and Perinatal Health, Bournemouth University, Bournemouth, UK.

**Keywords:** mental health illness, post-traumatic stress disorder, flood, India

## Abstract

Globally, frequent flooding causes higher magnitude of disaster among the developing and developed nations. Particularly, the Indian subcontinent is considered as highly vulnerable area for natural disaster and is affected most because of limited resources and coping strategies for post-disaster rehabilitation. Apart from the great impact on human health, floods have considerable impact on mental health. The most frequently diagnosed psychological illness in flood affected population is post-traumatic stress disorder (PTSD). In India, the incidence of PTSD in major natural disasters varies considerably depending upon the magnitude of event, with the highest rates reported of around 70%. Studies conducted during initial few months post-disaster, showed a higher occurrence of psychiatric manifestations. On the other hand, some reports suggested contrary results under similar circumstances. Notably, extreme age (children and elderly), female gender, socioeconomic status, pre-existing mental health issues and financial crisis post-disaster are the potential predisposing factors influencing the vulnerability of PTSD. In Indian context, the variability in the magnitude of psychiatric illness is mainly attributed to the ethnic diversity (vulnerable population), severity and type of flood event and social support. Still there is more to explore regarding the long-term sequelae of catastrophic floods on physical and mental trauma on disaster-affected populations.

## Introduction

Globally, serious flooding constitutes a major disaster in both rich and poor nations, we flooding reported in the United States of America, Europe, South East Asia, and Africa in the past decade [1]. In South Asia, the Indian subcontinent is the most vulnerable area for this natural disaster because of its geographic location and climatic conditions. The epidemiological data suggest that flooding is responsible for tremendous physical, social and psychological disruptions that necessitate extensive rehabilitation of the affected population. Apart from the great impact on human health due to injuries and unhygienic conditions, flooding can have considerable negative psychological effect on survivors in terms of mental illness, who can be diagnosed with post-traumatic stress disorder (PTSD), anxiety and depression [1,2]. Some studies have reported long-term effects on mental health with profound psychiatric morbidity in flood-affected individuals. The most frequently diagnosed psychological illness of people living in flood-affected areas is PTSD which manifests as difficulty in sleeping, emotional distress, avoidance and emotional arousal [2].

### Disaster-related mental health illness in India

The literature suggested an increase in the prevalence and symptomatology of PTSD among disaster-affected population in India. Many investigators have estimated the prevalence of PTSD in different natural calamities such as cyclones [3,4,5], tsunami [6,7], earthquakes [8,9,10], and fires [11]. Based on earlier Indian studies, the incidence of PTSD in major natural disaster varies considerably depending upon the magnitude of event, with the highest rates reported of around 70% [3-19].

Other studies from India, conducted in the first few months after a disaster, showed higher occurrence of psychiatric manifestations suggestive of PTSD symptoms. For instance, disaster studies after the super-cyclone Orrisa [3], the Gujarat earthquake [8], the Tamil Nadu tsunami [6], and the Bihar flood [12] reported a higher prevalence of PTSD among those who were diagnosed with studies found contrary results under similar circumstances [9,13]. Longitudinal studies focusing on mental illness demonstrated that the prevalence of PTSD start to decline with passage of time after the traumatic event. A study conducted by the Indian Council of Medical Research study [14] reported the frequency of PTSD to be 1.28% among earthquake survivors in Latur, Maharashtra after eighteen months of the incident which further decline to negligible five years after this disaster. Another study from Gujrat reported a much higher prevalence of PTSD (89.7%) during the first month after the earthquake, which slowly started to decline and reached 21% ten months post trauma [8].

Since there is a profound effect of flooding on the psychological wellbeing of the survivors, especially in low-income countries which have extremely limited coping strategies and resources for rehabilitation post disaster [15]. However, there is paucity of scientific evidence on the need for psychosocial support among the flood survivors in India.

### PTSD risk factors in India

Several studies from India have reported potential risk factors associated with the occurrence of PTSD among vulnerable populations following natural disasters. Children, adolescents and elderly are comparatively more vulnerable to mental illnesses when compared to adults post traumatic event. The first key risk factor is age, it is especially young and old age groups which are more vulnerable to PTSD in Indian context [3,16]. Furthermore, disaster-related mental illness manifestations such as anxiety, depression and PTSD are more evident in women than men, thus gender is also a risk factor. Many studies from India reported evidence of female vulnerability to mental health illness [7,16]. Studies from Tamil Nadu showed higher susceptibility of mental health issues such as PTSD among female post tsunami than male survivors. This is attributed to women’s social suppression in India’s patriarchal society. Another risk factor in India is socio-economic status as lower level of education and middle-class background were identified as the independent risk factors for the development of PTSD [3,4]. Financial crisis post disasters can also lead to mental strain and hence is considered as a risk factor for PTSD in India. Destruction of property and houses [4], physical injury to self [7,16], physical injury to family members [16], and death of family members [3,16] are also factors which might affect the likelihood of developing PTSD following a disaster. Also, there is an increased chance of developing PTSD post-disaster in survivors of major disaster with pre-existing mental health issues. Generally research in psychopathology suggests that PTSD is a neurobiological abnormality that is universal; however, the symptomatology may differ depending upon specific sociocultural practices.

### Impact of Floods on mental health in India

It is clearly important to deal with the acute psychological distress witnessed on a large scale early after traumatizing events through tailored and targeted psychological interventions. Investigators analyzing the relationship between exposure to flooding and mental health status have focused on understanding the underlying mechanisms or interventions which have potential benefits in regaining quality of life. Psychological support through disaster workers is one such mechanism which may contribute to psycho-rehabilitation of individuals exposed to flood. A recent cross sectional study from Kashmir investigated the impact of distinguished social support from family and relatives in adult survivors to look for correlation between exposure of flood and psychopathologic symptoms such as PTSD and depression [17]. The study suggested that greater support from family and friends decreases the negative psychological impact (PTSD & depression) after flood-exposure whereas less family support increased the vulnerability to mental illness. It has been suggested that social support facilitates a positive interactions that help to boost the interpersonal environment to overcome the trauma-related emotions or depression. Therefore, it is important to promote adults’ social support to counter the negative psychological impact post natural disasters.

Ishikawa and colleagues investigated the association between sociocultural characteristics and psychiatric disorders post flooding in Ladakh [18]. The authors found that out of the 318 individuals surveyed only two (0.6%) were found to have PTSD. The survey revealed that the criteria for assessment for psychiatric illness (PTSD and major depressive disorders) in this region mainly influenced by certain local cultural factors. Therefore, the diagnostic criteria of mental ill health developed from western culture did not fit well with the psychiatric sequelae of disasters in Ladakh. It is suggested that the sociocultural factors and ethnic temperament are playing a major role in the suppression of the manifestation of psychiatric illness and stress-related disorders.

An earlier study by Telles and colleagues [12] assessed the risk of developing PTSD and depression among individuals affected by the 2008 flood in Bihar. The study included 1,689 people with direct exposure to the flood. It was identified that elderly were more vulnerable to develop psychiatric illness after this major disaster. Hence, planning strategies for disaster relief should focus on this vulnerable population. A randomized controlled trial (RCT) was conducted by the Telles and others [19] investigated the effects of yoga among the survivors of flood in Bihar one month after the event. The authors reported a significant decline in the self-rated sadness after giving a week-long yoga intervention, whilst, the control group without yoga practice demonstrated an increase in self-rated stress. The study concluded that yoga intervention have potential to minimize the feelings of sadness or depression and prevent raising anxiety in flood-affected individuals.

As such, knowledge on potentially traumatizing events and its impact on the general population is lacking from Kerala state of India which experienced devastating flooding in 2018. Considering the heavy toll of flooding among the Kerala population a community-based study using mixed-methods [20] has been designed by Asim and colleagues for the assessment of PTSD, anxiety and depression among the flood-affected population in Kerala. The questionnaire content validity and study design were assessed by several subject experts participating in the International Conference on Mixed Method Research (ICMMR 2018) held at Mahatma Gandhi University, Kerala, India. This research project will help to assist mental health service providers to increase the relevance and impact of current psychological activities in Kerala and to help to formulate therapeutic modules for individuals to better cope with stress. Moreover, a systematic review and meta-analysis is also being designed to look into the burden of PTSD and other psychiatric disorders which will help to understand the psychological impact among flood affected populations that remains under-estimated.

## Conclusion

The magnitude of psychiatric illness varies with ethnic diversity due to the facts related to the vulnerable population, severity and type of flood event and social support in the country. Moreover, there is a need for population-based studies to assess the mental health impact on flood affected individuals in Kerala in order to develop evidence-based early interventions. Still there is more to explore regarding the long-term sequelae of catastrophic floods resulting in and worsening physical and mental trauma on populations affected by natural disasters. Apart from developing counter measures against disasters, develop special relief services, it is also important to inform policy makers about the magnitude and prevalence of mental illness in the affected population.
